# Selective metabolic regulations by p53 mutant variants in pancreatic cancer

**DOI:** 10.1186/s13046-024-03232-3

**Published:** 2024-11-26

**Authors:** Sabrina Caporali, Alessio Butera, Alessia Ruzza, Carlotta Zampieri, Marina Bantula’, Sandra Scharsich, Anna-Katerina Ückert, Ivana Celardo, Ian U. Kouzel, Luigi Leanza, Andreas Gruber, Joan Montero, Angelo D’Alessandro, Thomas Brunner, Marcel Leist, Ivano Amelio

**Affiliations:** 1 Chair for Systems Toxicology, Department of Biology, University of Konstanz, Konstanz, Germany; 2 Department of Pathology, University of California San Diego School of Medicine, La Jolla, San Diego, CA USA; 3 Department of Biomedical Sciences, Universitat de Barcelona, Casanova 143, Barcelona, 08036 Spain; 4 Chair for in Vitro Toxicology and Biomedicine, University of Konstanz, Constance, Germany; 5 Applied Bioinformatic Group, University of Konstanz, Constance, Germany; 6 Department of Biology, University of Padua, Padua, Italy; 7 Networking Biomedical Research Center in Bioengineering, Biomaterials and Nanomedicine (CIBER-BBN), Madrid, 28029 Spain; 8 University of Colorado Anschutz Medical Campus, Aurora, CO 80045 USA; 9 Chair for in Biochemical Pharmacology, University of Konstanz, Constance, Germany

**Keywords:** Tumour suppressor, Gain-of-function, Cancer Metabolism, Anti-oxidant capacity

## Abstract

**Background:**

Approximately half of all human cancers harbour mutations in the p53 gene, leading to the generation of neomorphic p53 mutant proteins. These mutants can exert gain-of-function (GOF) effects, potentially promoting tumour progression. However, the clinical significance of p53 GOF mutations, as well as the selectivity of individual variants, remains controversial and unclear.

**Methods:**

To elucidate the metabolic regulations and molecular underpinnings associated with the specific p53^R270H^ and p53^R172H^ mutant variants (the mouse equivalents of human p53^R273H^ and p53^R175H^, respectively), we employed a comprehensive approach. This included integrating global metabolomic analysis with epigenomic and transcriptomic profiling in mouse pancreatic cancer cells. Additionally, we assessed metabolic parameters such as oxygen consumption rate and conducted analyses of proliferation and cell–cell competition to validate the biological impact of metabolic changes on pancreatic ductal adenocarcinoma (PDAC) phenotype. Our findings were further corroborated through analysis of clinical datasets from human cancer cohorts.

**Results:**

Our investigation revealed that the p53^R270H^ variant, but not p53^R172H^, sustains mitochondrial function and energy production while also influencing cellular antioxidant capacity. Conversely, p53^R172H^, while not affecting mitochondrial metabolism, attenuates the activation of pro-tumorigenic metabolic pathways such as the urea cycle. Thus, the two variants selectively control different metabolic pathways in pancreatic cancer cells. Mechanistically, p53^R270H^ induces alterations in the expression of genes associated with oxidative stress and reduction in mitochondrial respiration. In contrast, p53^R172H^ specifically impacts the expression levels of enzymes involved in the urea metabolism. However, our analysis of cell proliferation and cell competition suggested that the expression of either p53^R270H^ or p53^R172H^ does not influence confer any selective advantage to this cellular model in vitro. Furthermore, assessment of mitochondrial priming indicated that the p53^R270H^-driven mitochondrial effect does not alter cytochrome c release or the apoptotic propensity of pancreatic cancer cells.

**Conclusions:**

Our study elucidates the mutant-specific impact of p53^R270H^ and p53^R172H^ on metabolism of PDAC cancer cells, highlighting the need to shift from viewing p53 mutant variants as a homogeneous group of entities to a systematic assessment of each specific p53 mutant protein. Moreover, our finding underscores the importance of further exploring the significance of p53 mutant proteins using models that more accurately reflect tumor ecology.

**Supplementary Information:**

The online version contains supplementary material available at 10.1186/s13046-024-03232-3.

## Introduction

P53, a critical tumour suppressor transcription factor encoded by the *TP53* gene, plays a pivotal role in safeguarding genome integrity [1, 2]. Its functional significance lies in orchestrating cellular responses, including cell cycle arrest, DNA repair, apoptosis, or senescence, thereby determining the fate of cells in the face of diverse stress conditions [1, 3, 4]. Beyond its fundamental role in genome maintenance, p53 emerges as a central regulator of cellular metabolism, impacting key processes such as glucose, lipid, and nucleotide metabolism, as well as mitochondrial oxidative respiration. This multilayer control underscores p53 ability to regulate cellular fate [5, 6]. Approximately 50% of human cancers exhibit p53 inactivation, with the majority of cancer-associated p53 mutations being predominantly missense. These mutations typically cluster within hot-spot regions of the DNA-binding domain (DBD), resulting in the generation of neomorphic p53 mutant proteins [7]. Notably, these GOF mutants have been implicated in promoting tumorigenesis by enhancing glycolysis, lipid synthesis, and serine metabolism [6, 8]. Furthermore, they exert influence over the microenvironment, shaping it in a manner that significantly impacts the tumorigenic potential of cancer cells [9–11]. However, the significance of the p53 mutant GOF has been also questioned by observation that depletion of p53 mutant proteins does not affect proliferation and cell death of several cancer cell lines [12]. These highlight the complexity of p53 mutations and shift attention over the possibility that they exhibit strong context-dependent behaviour [11].

Mutations within the *TP53* gene are frequently observed in pancreatic adenocarcinoma (PDAC), a globally pervasive and highly lethal malignancy characterized by an ominous prognosis and a 5-year survival rate below 10% [13, 14]. Specifically, the p53^R273H^ variant (whose mouse orthologue is the p53^R270H^) is among the most frequent p53 mutations, followed by p53^R175H^ variant (whose mouse orthologue is the p53^R172H^) observed in PDAC [15]. The ability of PDAC cells to grow in hostile conditions is supported by an extensive metabolic reprogramming [16], a well-established cancer hallmark, which is indispensable to help to sustain cancer cell survival and growth under microenvironmental stress conditions. Generally, the rewiring of metabolic pathways is a consequence of the activation of oncogenic signals and inactivation of tumour suppressor genes, including p53 [5, 17]. Despite the insights on the consequence of the loss of wild type p53 on cell metabolism, the impact of mutant p53 variants on the metabolic processes of pancreatic cancer remains not well understood, and the potential implications of these regulatory alterations in tumorigenesis are not fully elucidated.

In this study, we employed an integrative approach, combining global metabolomics, epigenomics, and transcriptomics with functional mitochondrial studies. Our findings reveal that the mutant p53 variants, R270H and R172H, exert very specific and selective effects on distinct metabolic pathways. While p53^R270H^ influences the mitochondrial tricarboxylic acid (TCA) cycle and glutathione metabolism, p53^R172H^ leads to an unexpected attenuation of the urea cycle metabolism, with potential tumour-suppressive implications. At the molecular level, p53^R270H^ and p53^R172H^ also exert specific and selective transcriptional and epigenetic effects. p53^R270H^ promotes the regulation of genes implicated in maintaining physiological mitochondrial fitness. In contrast, p53^R172H^ influences the expression of enzymes involved in urea cycle metabolism. Thus, we identify potential novel gain-of-function selective mechanisms of different p53 mutant variants on cell metabolism. These results emphasize the importance of conducting a thorough evaluation tailored to the specific mutation, context, and characteristics of p53 GOF mutants in order to comprehensively explore their clinical potential.

## Results

### p53^R270H^, but not p53^R172H^, affects tricarboxylic acid cycle and mitochondrial metabolism

**Fig. 1:**
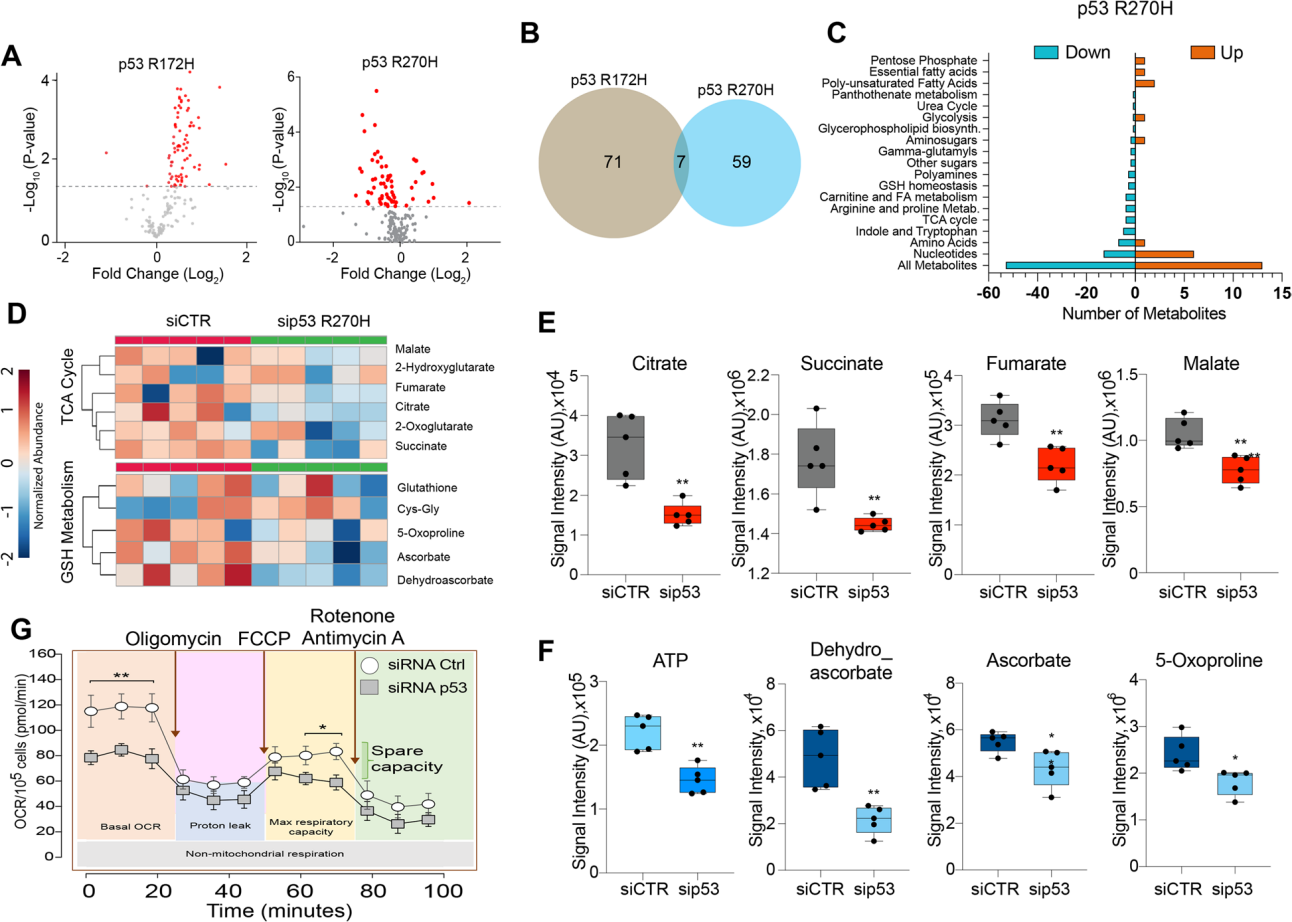
Metabolic profile of p53 mutant variants in pancreatic cancer. **A **Volcano Plot of differentially modulated metabolites related to all biological pathways upon p53^R270H^ depletion in KPC^R270H^ cells (right plot) or p53^R172H^ depletion in KPC^R172H^ cells (left plot). **B **Venn diagram indicates no significant overlap between p53^R270H^- and p53^R172H^-dependent metabolomes. **C** Modulated metabolic pathways related to p53^R270H^ depletion. **D** Heat maps representing deregulated metabolites belonging to TCA cycle and GSH Metabolism upon p53^R270H^ depletion in KPC^R270H^ cells. **E**, **F** Box-plots of all significantly up- or down-regulated metabolites upon mutant depletion in KPC^R270H^ cells. Metabolites abundancies are represented as signal intensities (AU: Arbitrary Units). *P*-values= * *p* < 0.05; ** *p* < 0.01. Metabolomic analysis was performed on 5 biological replicates for each condition. **G **Seahorse mitochondrial flux analysis performed in KPC^R270H^ and p53-depleted KPC^R270H^ cells after 72h of silencing. Measurements were performed following injection of oligomycin (complex V inhibitor, 1 μM), FCCP (protonophore, 1.5 μM) or a mix of rotenone (complex I inhibitor, 0.5 μM) and antimycin (complex III inhibitor, 0.5 μM) to analyse respectively ATP generation, maximal respiratory capacity, and spare capacity. OCR was quantified by normalizing to the number of H-33342 positive cells. *P* -values= * *p* < 0.05; ** *p* < 0.01. *N*= 3 ± SEM

Metabolic reprogramming in cancer provides substrates to support cell proliferation, migration, invasion and survival capabilities at different stages of carcinogenesis [18]. We selected PDAC cell line derived from pdx1-CRE mouse models with pancreas-specific expression of constitutively active KRAS (LSL-KRAS^G12D^) and the mutation p53^R270H^ (named KPC^R270H^) or p53^R172H^ (named KPC^R172H^). To identify the metabolic changes associated to the p53^R270H^ and p53^R172H^ mutant variants, we performed a global metabolomic analysis in KPC^R270H^ and KPC^R172H^ proficient- and deficient- pancreatic cancer cells. The level of several metabolites belonging to nucleotides, amino acids and energy metabolism pathways were significantly affected by depletion of both variants underlying an effective impact of this neomorphic proteins on cellular processes (Fig. 1A, Supp. Fig. 1A). Integration of both datasets, however, indicated that only 7 metabolites are changing toward the same direction in both genotypes (Fig. 1B), thus mutant p53 variants associated metabolome emerge to be highly specific.

KPC^R270H^ metabolome revealed significant decrease in levels of tricarboxylic acid cycle (TCA) intermediates, such as citrate, succinate, fumarate and malate, suggesting an effect of the mutant p53^R270H^ on mitochondrial metabolism and consequent ATP level (Fig. 1C-F). Moreover, our metabolomic analysis identified also deregulations in the glutathione (GSH) metabolic pathway (Fig. 1D, F). We observed significant reduction in PDAC cells depleted of p53^R270H^ of 5-oxoproline, ascorbate and dehydroascorbate, compounds involved in the redox homeostasis [19]. The balance of antioxidant levels is a key aspect for cancer cell. Reactive oxygen species (ROS) accumulation is frequently occurring in cancer cells as a consequence of mitochondrial dysfunctions.

To corroborate our metabolomic analysis indicating impairment of mitochondrial fitness, next we tested mitochondria function by measuring the oxygen consumption rate (OCR) with a Seahorse stress test. Under basal conditions, p53^R270H^-depleted KPC cells displayed a reduced OCR compared to KPC-expressing p53^R270H^ (Fig. 1G). Following addition of carbonyl cyanide 4-(trifluoromethoxy)phenylhydrazone (FCCP), that uncouples oxygen consumption from ATP-synthesis, we could not detect any major increase in OCR to a maximum level suggesting a general low spare capacity in our KPC cell line. Nevertheless, we revealed a significant general reduction in the rate of maximal respiration in p53^R270H^ depleted cells (Fig. 1G, Supp. Fig. 1C). To comprehensively assess mitochondrial fitness, we measured mitochondrial membrane potential using JC-1 staining followed by FACS analysis. Silencing of p53^R270H^ altered mitochondrial membrane potential, showing an increase in the J-aggregate signal, which we interpreted as indicative of stressed mitochondrial activity (Supp. Fig. 1B).

Our data indicate a functional link between mutant p53^R270H^, mitochondrial metabolism, GSH homeostasis and general mitochondrial fitness that could influence cancer cell fate [20]. Reliance on oxidative phosphorylation has been reported to be a key metabolic feature of Ras-driven PDAC stem-like cells that leads to tumour recurrence [21–23], our data however indicate a link between mutant p53^R270H^ and these metabolic processes of possible relevance for pancreatic cancer biology.

### p53^R172H^ affects the urea cycle

**Fig. 2:**
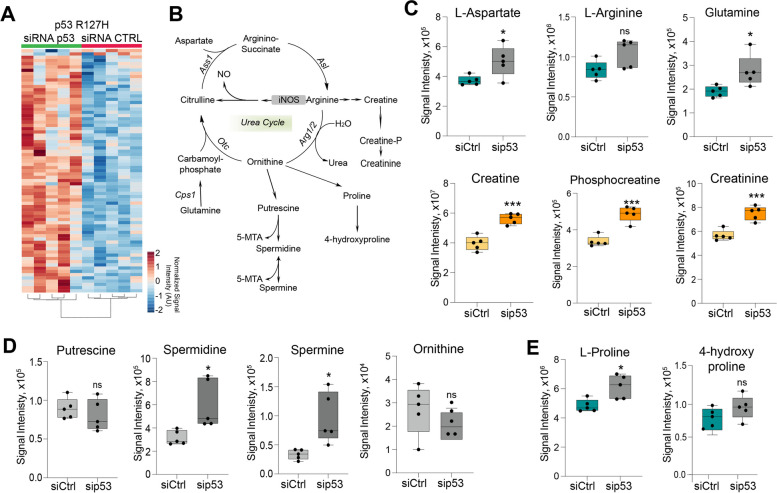
p53^R172H^ -deficient cells display dysregulation of urea cycle pathway. **A** Heatmap of global metabolic changes following depletion of p53^R172H^ mutant variant. *p* value <0.05 and a -1.3<fold change<1.3 were used as threshold. Metabolite abundancies are shown as Signal Intensity AU (Arbitrary Units). *N*=5 biological replicates. **B** Graphical representation of Urea Cycle metabolic pathway. Cps1: carboamylphosphate 1; Otc: Ornithine transcarbamylase; Ass1: Arginosuccinate synthase 1; Asl: Arginino-succinate lyase; Arg1/2: Arginase 1/2; CkB: Creatin kinase B; iNOS: inducible Nitric oxide synthase; NO: Nitric oxide. **C**-**E** Box-plots of urea cycle related metabolites in mouse pancreatic ductal carcinoma (PDAC) cells following p53^R172H^ mutant variant depletion. *N*=5 biological replicates per condition. Graphs are shown as mean ± SD. * *p* value <0.05; ** *p* value <0.01; *** *p* value <0.001; ns *p* value not significant

While p53^R172H^ did not influence TCA cycle and anti-oxidant metabolism, the top 25-enriched metabolites influenced by this p53 mutant variant appeared to belong to arginine and proline metabolism, aspartate metabolism, ammonia recycling and urea cycle (Fig. 2A-E). Urea cycle has recently emerged as a critical metabolic process for cancer cells [24]. Furthermore, wild-type p53 prevents ureagenesis and influences ammonia metabolism, promoting resilience against toxic ammonia upon loss of wt p53 function. These wt p53 metabolic changes also promote polyamine biosynthesis and enhances cell proliferation [25]. We observed that depletion of p53^R172H^ significantly increased the levels of L-aspartate, L-glutamine, creatine, phosphocreatine, creatinine, proline, putrescine, spermidine and spermine (Fig. 2C-E), that are related to pro-tumorigenic mechanisms, including alterations in energy production [26] and polyamines biosynthesis [27].

Urea cycle can influence synthesis of pyrimidines, essential substrates for DNA synthesis and thus proliferation. We observed a significant increase in the entire pool of nucleotide triphosphates and cofactors, including both purines and pyrimidines (Supp. Fig. 2). Hence, the metabolic changes associated to the p53^R172H^ appear to significantly impact general synthesis or consumption of nucleotides. Overall, our data show that p53^R172H^ has a specific role in controlling the metabolome of pancreatic cancer cells, affecting urea cycle pathway.

### p53^R270H^ impacts expression of genes involved in mitochondrial fitness

**Fig. 3:**
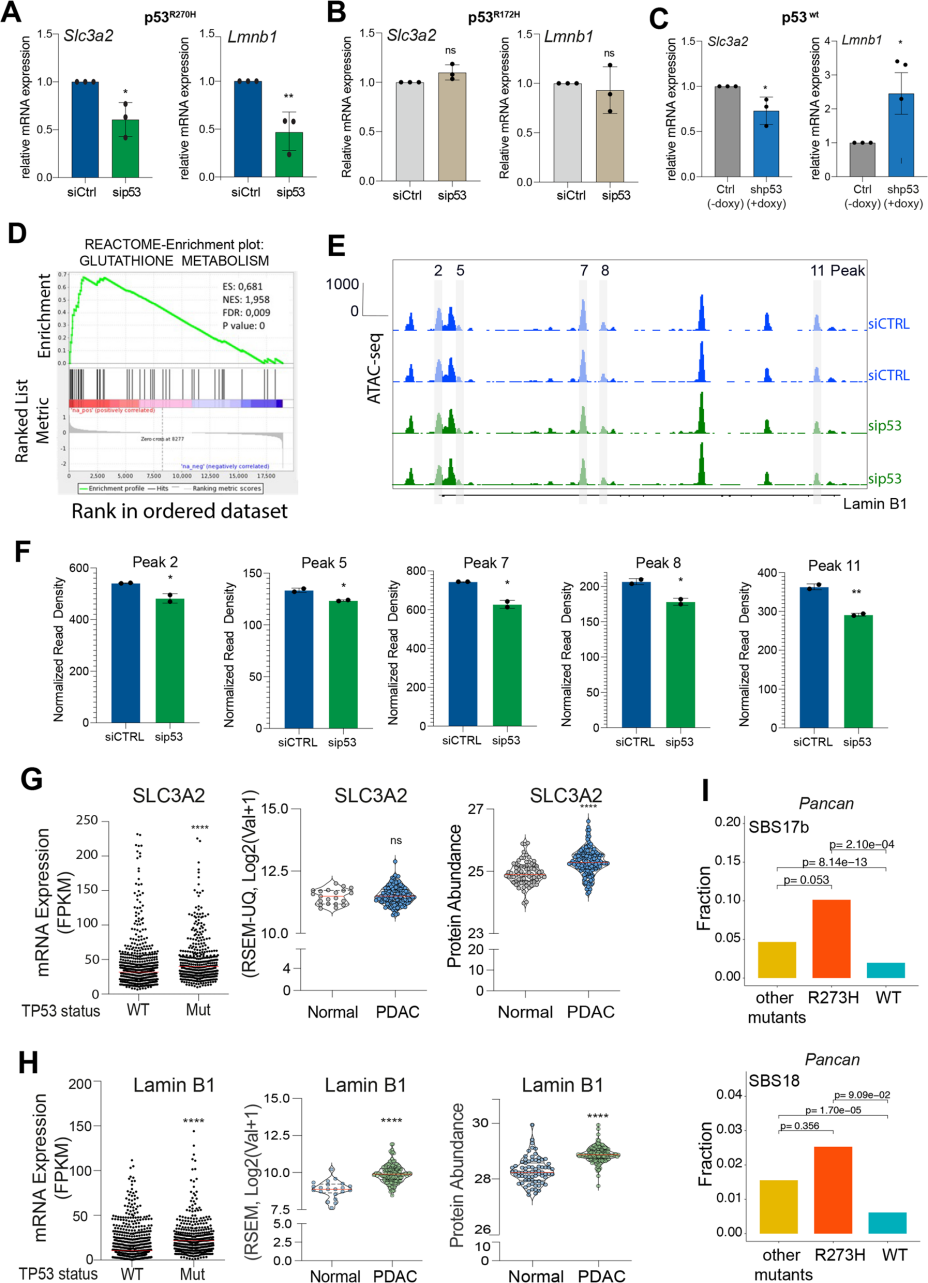
p53^R270H^ dictates a specific gene expression programme. **A-C** RT-qPCR displays the mRNA levels of Slc3a2 and Lamin B1 following in p53^R270H ^(A), p53^R172H^ (B) and p53^WT^ (C) proficient-/deficient-cells. Graphs are shown as mean ± SD of 3 biological replicates. * *p* value <0.05; ** *p* value <0.01. **D **Reactome-enrichment plots showing “Glutathione metabolism” as one of the top enriched pathways upon mutant p53^R270H ^depletion in KPC^R270H^ cells. **E**, **F** ATAC-seq profile showing changes in chromatin accessibility in Lamin B1 gene following p53^R270H ^depletion. Bar-plots show ATAC-seq peaks significantly modulated following p53^R270H ^silencing. T-test analysis was performed. *P*-values= * *p* < 0.05; ** *p* < 0.01, **** *P* < 0.0001, ns *p* value not significant. **G**, **H** Scatter dot plot representing Slc3a2 and Lamin B1 expression levels in all p53 mutated tumours compared to the counterpart p53 wt tumours. Patient data were processed and retrieved by TCGA PanCancer Atlas from c-BioPortal database. Scatter dot plot showing Lamin B1 and Slc3a2 expression and protein levels in pancreatic ductal adenocarcinoma tissues compared to the adjacent normal tissues by the analysis of published proteogenomic date [35]. *P*-values= * *p* < 0.05; ** *p* < 0.01, **** *P* < 0.0001. **I** Fraction of tumours in which the SBS17b or the SBS18 mutational signature was present stratified by p53 mutation status: p53^R273H^ expressing-tumours, tumours expressing p53 with other pathogenic mutations and p53 WT tumours. *p* values are indicated

We next asked how the different mutant p53 variants could promote the observed metabolic changes. To this end, we firstly analysed transcriptomic and epigenomic global profiling of p53^R270H^ proficient and deficient KPC cells. p53^R270H^ appeared able to affect a variety of pathways connected to redox homeostasis, as revealed by a gene set enrichment analysis (GSEA) (Fig. 3D and Fig. Supp. 3A). We validated by RT-qPCR two genes involved in redox homeostasis and in mitochondrial regulation whose expression is altered in a p53^R270H^-dependent manner. We found that mutant p53^R270H^ depletion negatively affects the transcription level of Solute Ca﻿rrier Family 3 Member 2 (*Slc3a2*) and Lamin B1 (*Lmnb1*) genes. These genes were selectively regulated by mutant p53^R270H^, but not by mutant p53^R172H^ (Fig. 3A, B). The Slc3a2 gene encodes a transmembrane glycoprotein that forms heterodimers with membrane transport proteins, such as Slc7a11, to constitute the Xc- system, or Slc7a5 to constitute the L-type amino acid transporter 1 (LAT1). The Xc- system plays a crucial role in regulating the exchange of extracellular cystine and intracellular glutamate, thereby contributing to the synthesis of glutathione [28, 29], while LAT1 is important for amino acid uptake under condition of nutrient deprivation [30]. Recently, LAT1 was implicated in the control of serine and glycine metabolism by p53 mutant [8]. Lamin B1 is a component of nuclear lamina which sustains nuclear architecture and organization as well as affects the activity of chromatin-modifying enzymes and gene transcription [31]. Lamin B1 is not directly implicated in mitochondrial metabolism, but lamins have been found to be relevant for the crosstalk between oxidative stress, metabolic regulations and nuclear functions [32]. Lamin A/C impairment has been associated to an altered chromatin landscape and gene expression, impacting on oxidative stress and mitochondria [33]. Similarly, Lamin B1 was shown to control oxidative stress response via mechanisms of sequestration of transcriptional factors, that in turn impact regulation of gene expression [34]. Hence, Lamin B1 appears to be potential upstream molecular hub, dictating gene expression regulations, responsible for the metabolic effects observed in p53^R270H^. We therefore verified whether the increased Lamin B1 mRNA levels were linked to p53 GOF chromatin regulations. Transposase-accessible chromatin followed by deep sequencing (ATAC-seq) data indicated that p53^R270H^ depletion was associated to significant changes in chromatin accessibility upstream (peak 2) and in the intergenic regions (peak 5, 7, 8 and 11) of Lamin B1 gene (Fig. 3E, F).

To verify whether the p53^R270H^ -/Slc3a2 -/Lamin B1 axes were also observed in human cancers, we performed an analysis of cancer genomic and transcriptomic data on the TCGA Pan-Cancer Atlas. Intriguingly, p53-mutant tumours displayed significant higher levels of both Lamin B1 and Slc3a2 genes as compared to wild-type-p53 cohort, thus supporting our experimental data that indicate an effect of p53 mutant on Lamin B1 and Slc3a2 expression levels (Fig. 3G, H). Moreover, by analysing transcriptomics and proteomics datasets of human PDAC [35], we found that Slc3a2 and Lamin B1 were significantly elevated in PDAC tissue when compared to the normal tissue counterpart, thus indicating a positive role of these genes in pancreatic cancer progression (Fig. 3G, H).

As described above, p53^R270H^ depletion leads to mitochondrial dysfunction, with potential implications for the oxidative redox homeostasis (Fig. 1). ROS can directly produce mutagenic processes, thus contributing to genome instability and cancer evolution [36]. Therefore, we investigated whether p53^R273H^ mutant expression determined specific patterns of somatic mutations across human cancers. Screening for enrichment in mutational signatures in the TCGA cohort we found several single base substitution (SBS)-mutational signatures associated to p53 mutant status (Supp. Fig. 3C), among which we revealed the frequently observed SBS2/SBS13 (APOBEC signatures) and SBS1/SBS5 (age-related signatures). Moreover, in support of our experimental data the mutational signatures associated to ROS-mediated genotoxicity, SBS17b/SBS18 [37], were also significantly enriched in p53 mutant tumours (Fig. 3I). This suggests that these tumours evolve under increased ROS level and indicate with correlative evidence that p53 mutant status might have functional relationship with redox homeostasis.

### p53^R172H^ mutant impacts expression of gene involved in the urea cycle metabolism

**Fig. 4:**
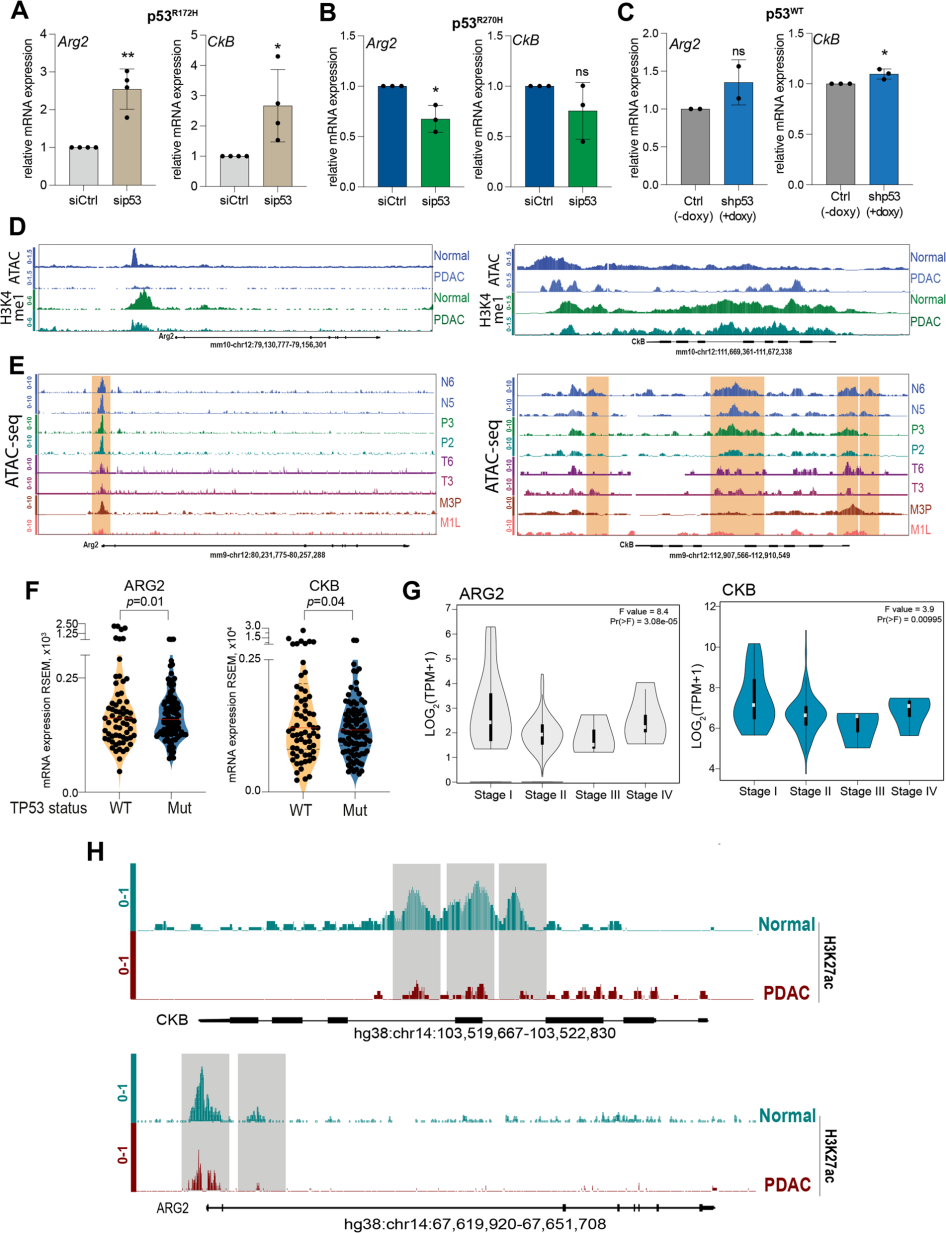
Arginase 2 and Creatin Kinase are regulated by p53^R172H^. **A**-**C** Arginase 2 (Arg2) and Creatin Kinase B (CkB) mRNA expression level by RT-qPCR in mouse PDAC p53^R172H^ (left), p53^R270H^ (middle), p53^WT^ (right) proficient-/deficient-cells. Graphs are shown as mean ± SD of at least 2 biological replicates. ns: not significant, * *p* value <0.05; ** *p* value <0.01. **D **ATAC-seq and ChIP-seq tracks for H3K4me1 showing gene loci of Arg2 and CkB in pancreatic ductal carcinoma (PDAC) and normal pancreatic murine tissues. **E** ATAC-seq tracks showing gene regions of Arg2 and CkB across PDAC stages. N6, N5: Mouse normal pancreatic organoids; P3, P2: Mouse PanIN organoids: T6, T3: Mouse PDA tumor organoids, M3P: Metastasis Peritoneum, M1L: Metastasis Liver. *N*=2 replicates. **F** ARG2 and CKB mRNA expression in the cohort of human pancreatic adenocarcinoma patients (TCGA, PDAC, PanCancer) stratified on the basis of p53 status. **G** Stage plot of ARG2 (left panel) and CKB (right panel) mRNA expression across the different stages (I-IV) of pancreatic adenocarcinoma patients. Source: GEPIA. **H **H3K27ac ChIP-seq tracks for gene regions of ARG2 and CKB in PDAC and normal pancreatic human tissue

Enzymes involved in the urea cycle are frequently deregulated in cancer [38]. To investigate whether the metabolic changes in p53^R172H^-deficient cells were associated to alterations in the gene expression of these enzymes, we performed a gene expression analysis by RT-qPCR. Our data revealed a significant and consistent upregulation of Arginase 2 (Arg2) and Creatin Kinase B (CkB) in mouse PDAC p53^R172H^ deficient-cells compared to mouse PDAC p53^R172H^ proficient-cells (Fig. 4A). Notably, upregulation of Arg2 and CkB was specifically observed upon p53^R172H^ manipulation, while modulation of the p53^R270H^ mutant and wt p53 did not influence the expression level of the two enzymes (Fig. 4B, C). Particularly, our data show a modest downregulation of Arg2 in p53^R270H^ deficient-cells and no significant alterations in wt p53 deficient-cells. Thus, at the molecular level we show that impact on the expression of Arg2 gene represents a specific consequence of p53^R172H^ mutant function, that might be responsible for the observed changes in the urea cycle metabolites. The impact of p53^R172H^ on CkB expression also appeared very intriguing: creatine kinases have energy buffering role, sustaining high ATP/ADP ratios, by reversibly transfer of a phosphoryl group from ATP to creatine [39]. CkB is a target of mechanosensitive transcriptional factor YAP, that in response to stiffness of PDAC extracellular matrix promotes phosphocreatine production and metastasis [26]. As a crosstalk between p53 mutants and YAP exists [40–42], at the mechanistic level this could account for the observed p53^R172H^-dependent regulation of CkB.

To investigate whether the observed alterations in mRNA levels of *Arg2* and *CkB* were associated to changes in epigenetic alterations occurring during PDAC progression, we performed extensive analysis of publicly available ChIP-seq and ATAC-seq data from the ChIP-Atlas database comparing PDAC and normal murine pancreatic tissues. In normal murine pancreas, we observed the presence of a region with higher chromatin accessibility corresponding to the Transcription Start Site (TSS) of *Arg2*, which is also enriched for the markers of active euchromatin mono-methylation of lysine 4 of histone H3 (H3K4me1). Consistently, in PDAC murine tissues we observed lower levels of chromatin accessibility showed by ATAC-seq peaks (Fig. 4D). Analysis of the CkB genomic locus revealed a similar pattern of permissive histone modifications, such as H3K4me1 with a slightly higher chromatin accessibility around the TSS but lower along the gene body in mouse PDAC tissue. Most importantly, at the 3’-untranslated region (3’-UTR) we observed a highly accessible area, enriched in H3K4me1 modification (Fig. 4D), which could represent a downstream regulatory region responsible for the higher expression of CkB in normal murine pancreatic tissues.

To ascertain whether these transcriptional changes could be observed during the pancreatic cancer progression, we investigated the accessibility of the genomic loci of interest by taking advantage of public available recently generated data [43]. The authors employed an organoid culture model of PDAC, which preserves the biological features of normal, PanIN, primary tumor, and metastatic lesions. We selected two representative samples for normal pancreatic ducts (hereafter referred to as N5 and N6), PanIN lesions (hereafter referred to as P3 and P2), autochthonous primary tumors (T6 and T3) and paired metastatic lesions from the peritoneum or the liver (M3P and M1L). Analysis of both the Arg2 and CkB genomic loci showed a remodeling of the chromatin accessibility, characterized by a clear reduction in the transition from normal tissue to primary tumors, followed by an increase in paired metastases (Fig. 4E).

To test whether the p53^R172H^/Arg2 and p53^R172H^/CkB axes were also conserved in human cancers, we conducted a bioinformatic analysis of a PDAC cohort belonging to the TCGA PanCancer Atlas dataset. Particularly, we sought to determine the levels of Arg2 and CkB in relation to p53 mutational status. Interestingly, mutations in p53 were significantly associated with lower levels of Arg2 and CkB (Fig. 4F), highlighting the biological relevance of p53^R172H^/Arg2 and p53^R172H^/CkB axes in the pathogenesis of PDAC. Furthermore, to extend our analysis, we focused on the prognostic significance of Arg2 and CkB expression. With the help of the Gene expression Profiling Interactive Analysis (GEPIA), we observed that the expression levels of the indicated genes underwent a decrease through the different stages of pancreatic adenocarcinoma (PAAD) **(**Fig. 4G).

Given the biological relevance of Arg2 and CkB in the pathogenesis and prognosis of human PDAC, we investigate whether the epigenetic modifications described above in mouse models are conserved in human tissues. We compared available ChIP-seq and ATAC-seq data of PDAC and normal human pancreatic tissues. Analysis of both Arg2 and CkB genomic loci revealed a reduction of the acetylation of lysine 27 of histone H3 (H3K27ac), a histone marker indicating a permissive status of the chromatin, in cancer tissues as compared to the normal counterpart (Fig. 4H). These data might confirm the epigenetic remodelling observed in mouse models and suggest the presence of a conserved mechanism for the regulations of Arg2 and CkB expression in mice and humans*.*

Overall, these data show that Arg2 and CkB expression is influenced by p53^R172H^ and their epigenetic and chromatin accessibility change across PDAC progression, supporting a role of these enzymes in the process of tumorigenesis.

### p53 mutant variants do not produce selective advantages to cancer cells

**Fig. 5:**
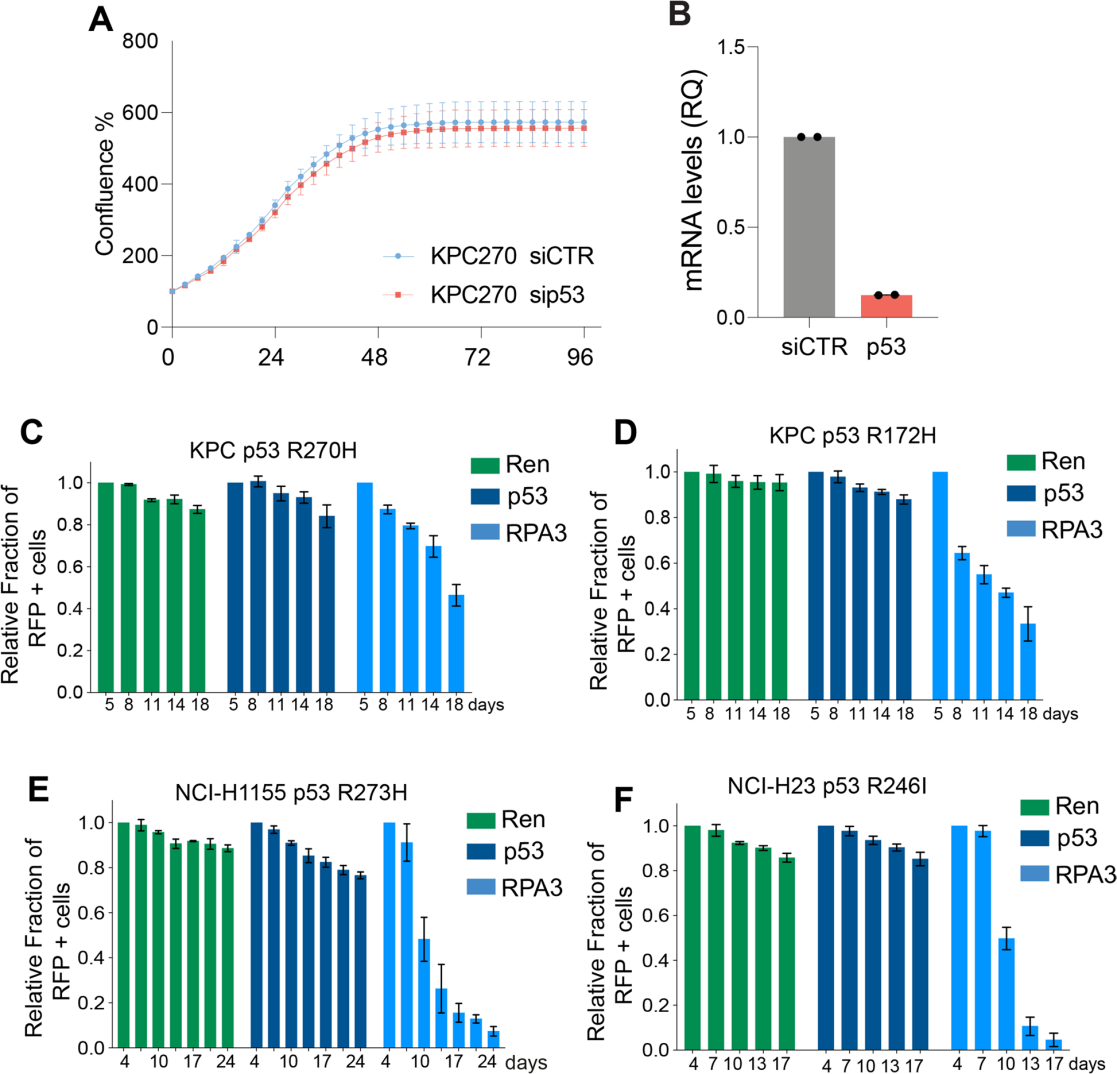
p53 mutant variants do not confer selective advantages *in vitro. ***A**-**B **Incucyte time-lapse analysis reports growth pattern of p53^R270H ^proficient and deficient PDAC KPC cells. RT-qPCR (B) indicating silencing efficiency of p53^R270H ^in the experiment shown in A. **C**-**F** Cell competition assay based on FACS measurement of RFP+ population (p53 mutant depleted) in different p53 mutant carrying cancer cell lines at different time points (up to 18 or 24 days culture). gRNA against Renilla (Ren) and RPA3 were used as negative and positive control respectively. Bars report mean +/- SD of three independent biological replicates

We next decided to investigate the influence of p53^R270H^ on cellular proliferation to determine the implications of the described metabolic regulations. Our goal was to assess whether p53^R270H^ or p53^R172H^ expression conferred a growth advantage or disadvantage to PDAC KPC cells. Initially, we conducted short-term analyses of cellular proliferation in cells with and without p53^R270H^. Time-lapse imaging using IncuCyte analysis was employed in cells transfected with control siRNA (siRNA CTRL) and siRNA targeting p53 (siRNA p53). Within 48 h, KPC cells reached confluence with no discernible differences observed (Fig. 5A, B). Next, we investigated whether p53^R270H^ or p53^R172H^ expression influenced long-term proliferation, potentially imparting a selective advantage over isogenic cell counterparts lacking p53 expression. To achieve this, we utilized a CRISPR/Cas9 approach to target p53 and assessed cell competition with a subpopulation of cells transduced with a non-targeting gRNA. Our findings revealed that p53^R270H^ or p53^R172H^ expression had no discernible impact (Fig. 5C, D), suggesting an absence of gain-of-function effects under these conditions. To further validate and generalize this finding, we expanded the cell competition assay to additional p53 mutant-carrying cell lines. This included a human cell line carrying the orthologous p53^R270H^ variant, p53^R273H^ (NCI-H1155), and another human cell line with the commonly observed human p53 variant, R246I (NCI-H23). CRISPR/Cas9 deletion of the endogenous p53 mutant did not confer any disadvantage or advantage to any tested cell line (Fig. 5E, F). While unexpected, this finding aligns with recent observations in other cell lines that several p53 mutant variants do not confer a selective advantage to cancer cells [12].

**Fig. 6:**
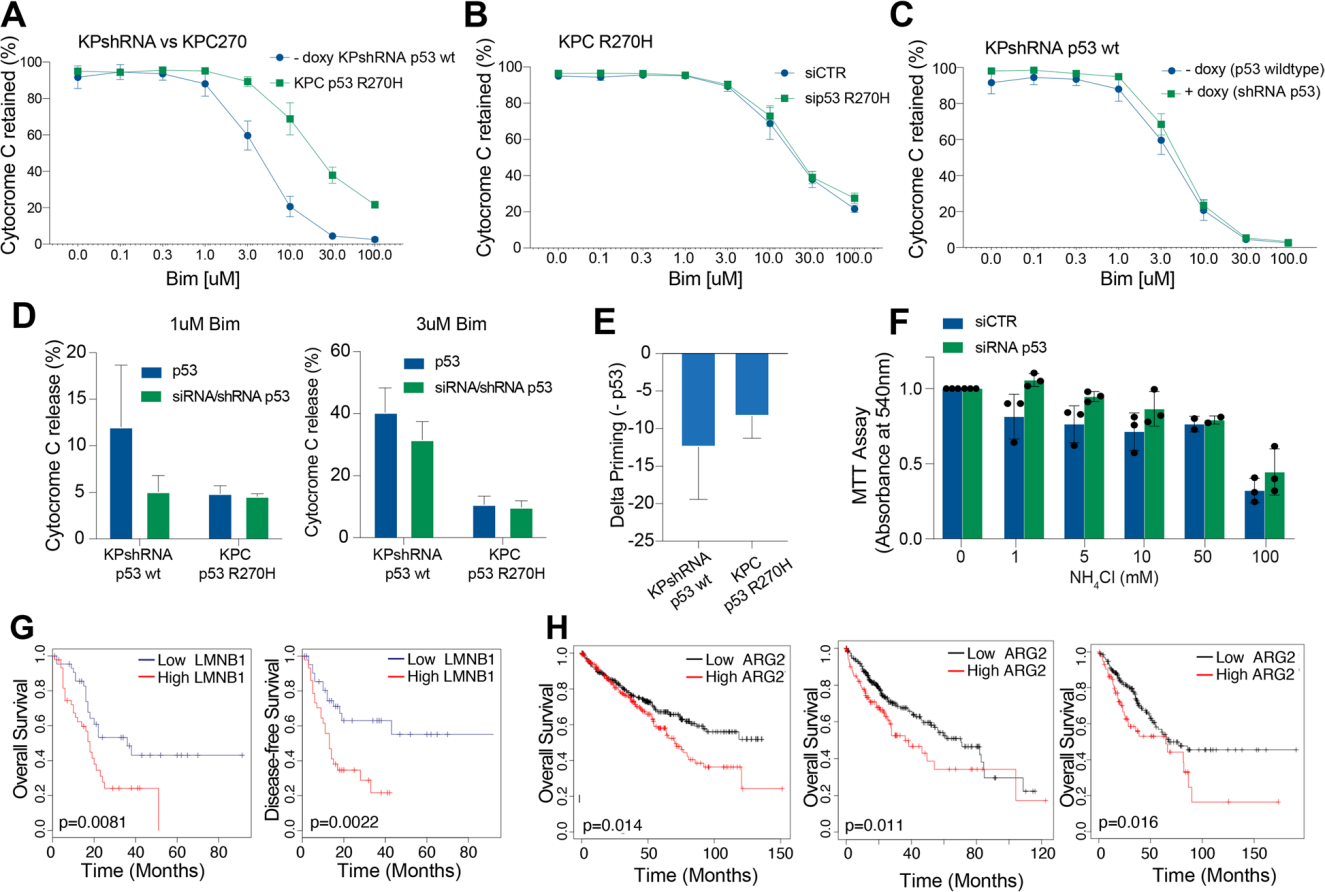
p53 mutant variants do not alter cell fitness in vitro, but patients’ prognosis is altered by p53 mutant-related gene networks. **A-E **BH3 profiling conducted by measurement of Cytochrome-C release at increasing doses of Bim in pancreatic cancer cells with different p53 status following p53 depletion.** F **Viability analysis conducted on p53^R172H^ proficient (siCTR transfected) and deficient (p53 siRNA transfected) KPC^R172H^ cells exposed for 24h to increasing doses of ammonia.** G** Overall-survival and disease-free survival Kaplan-Meier curves by stratifying PDAC patients from TCGA according to Lamin B1 mRNA transcription levels. **H **Kaplan-Meier overall survival curve for ARG2 expression level (high vs low) in different cohorts of cancer patients (kidney, left panel; liver, central panel; sarcoma, right panel). *P* values are indicated in the panels

Next, we explored whether the metabolic regulations of p53 mutant variants might confer selective advantages in response to specific stressors. The impact of the p53^R270H^ variant on mitochondrial metabolism suggests it may affect mitochondrial fitness and consequently alter the priming of the intrinsic apoptotic pathway (mitochondrial apoptosis) [44, 45]. Altered susceptibility to mitochondrial apoptosis could indeed correlate with the p53-mediated chemoresistance phenotype. To investigate this, we conducted BH3 profiling, analysing cytochrome C release in response to increasing doses of the BH3-only protein, Bim [46, 47]. As expected, mouse PDAC cells expressing wild-type p53 (KPshRNA) were significantly more prone to apoptosis compared to KPC270 cells [48], which express p53^R270H^ (Fig. 6A). Converting p53^R270H^-expressing cells to a p53-deficient status through siRNA-mediated depletion of p53 did not produce any significant effect on apoptotic priming (Fig. 6B-E). Thus, the expression of p53^R270H^ does not appear to exert GOF effects on the early steps of mitochondrial apoptosis.

The p53^R172H^ variant seems to regulate urea cycle metabolism (Fig. 2). The urea cycle is known to enhance cancer cell metabolism by promoting resilience against ammonia accumulation and facilitating polyamine synthesis, which can spur proliferation [24]. To determine whether p53^R172H^ plays a role in these processes and thereby impacts tumorigenesis, we investigated whether its depletion alters the sensitivity of KPC cells to ammonia. By exposing cells to increasing ammonia concentrations, we evaluated the general cell viability of p53^R172H^-proficient and p53^R172H^-deficient cells (following sip53 transfection). Despite escalating ammonia levels progressively diminishing viability, no significant changes were observed with or without p53^R172H^ expression (Fig. 6F). Hence, the expression of p53^R172H^ does not appear to retain any tumor-suppressive properties of wild-type p53 nor exert GOF effects under the tested experimental conditions.

### p53 mutant downstream effectors have prognostic implications for cancer patients

To further test the significance of our described molecular mechanisms and associated metabolic regulations, we verified whether these have any significance in a clinical setting as prognostic indicators. p53 inactivation is, as a matter of fact, a negative prognostic factor in several cancers [49]. However, whether the effect is exclusively related to the loss of the wild-type p53 expression or if any additional contribution can be attributed to the expression of mutant p53 protein variants remains difficult to assess due to the limitations of the patient cohort size when stratified for specific variants. Hence, we tested these questions by conducting Kaplan–Meier survival analysis, stratifying PDAC patients based on the downstream identified effectors. As downstream targets of p53^R270H^, we analysed Lamin B1 mRNA levels, which appeared as a poor prognostic factor, associated with low overall survival and a high probability of disease recurrence (Fig. 6G). Moreover, consistently with our original hypothesis, a survival analysis showed that higher levels of the p53^R172H^ target Arginase 2 were also associated with lower patient survival (Fig. 6H). Thus, we interpreted this data as an additional indication that p53 mutant status is frequently associated with cancer prognosis. The question remains open on the causative relationship for these events and on the possibility to generate these relationships perturbing relevant molecular signalling pathways in experimental models that accurately recapitulate the biology of a real tumour ecosystem.

## Discussion

Mutations in the p53 gene are the most common genomic alterations in cancer. Despite this, no effective therapeutic strategy currently exists to target p53 mutations [50]. The high frequency of p53 missense mutations suggests a selective advantage in accumulating mutant p53 proteins. However, whether and how these mutations confer a benefit to cancer cells remains controversial [51, 52]. Clarifying this relationship would offer valuable insights for developing cancer therapies. Specifically, approaches to either restore wild-type p53 function or to inhibit mutant p53 activity would require distinct therapeutic strategies.

In this study, we used a comprehensive approach that integrates global metabolomics, epigenomics, and transcriptomics in mouse pancreatic cancer cells with different p53 hotspot mutations to explore the mechanistic and functional consequences of p53 GOF effects in PDAC. Our investigation highlights the roles of the p53^R270H^ and p53^R172H^ mutants in regulating the metabolic processes of pancreatic cancer cells.

A key finding from our data is that these two p53 mutants exert markedly different metabolic effects, reflecting distinct mechanisms. p53^R270H^ appears to influence the expression of genes involved in mitochondrial metabolism and redox homeostasis, aligning with broader impacts on metabolic regulation and mitochondrial activity. In contrast, p53^R172H^ affects the urea cycle, a pathway used by cancer cells to manage ammonia clearance and adapt to challenging metabolic conditions. Notably, these mutants show no overlap in their regulation of cellular metabolism, even though they were studied in comparable experimental models. Both cellular systems were derived from isogenic mice engineered to develop pancreatic cancer lesions using pdx1-CRE mouse models with pancreas-specific expression of constitutively active KRAS (LSL-KRASG12D) and either p53^R270H^ (KPC^R270H^) or p53^R172H^ (KPC^R172H^). Additionally, p53 mutant expression was silenced using the same siRNA sequence for both mutants, ruling out off-target effects from the siRNAs as a cause for the observed outcomes.

Our study also reveals an intriguing observation: while metabolic analyses suggest a potential oncogenic role for p53^R270H^ and a retained tumor-suppressive capacity for p53^R172H^, these effects do not translate into altered cellular behaviors in our experimental system. This finding, in line with a recent report [12], raises questions about the biological significance of the observed metabolic changes. Thus, while there is ongoing debate about the oncogenic potential of p53 mutant GOF effects [11, 53–55], our results add complexity to this discourse, suggesting a need for further investigation into GOF mechanisms within models that more accurately reflect tumor ecology. In particular, future studies should explore the phenotypic impact of various p53 mutants, as selective advantages might only become evident in more appropriate in vivo conditions, potentially in the context of metastasis rather than primary tumor growth, where GOF effects were orignally described [56, 57].

## Conclusions

Understanding whether p53 mutant proteins are viable targets for cancer therapy remains a critical question in clinical oncology. Our findings underscore the importance of using appropriate experimental models to address this question comprehensively. Moreover, our data highlight the variant-selective effects of p53 mutations, urging a shift from viewing different p53 mutants as a single class toward a nuanced understanding of each mutant's specific roles in diverse contexts. Developing experimental approaches along these lines will clarify the therapeutic potential of targeting p53 mutant proteins and guide future treatment strategies.

## Materials and methods

### Cell culture and siRNA transfection

KPC 172 and KPC 270 cells derive from PDAC that developed in a *Pdx1-Cre; LSL-Kras*^*G12D/*+^*; LSL-Trp53*^*R172H/−*^* or LSL-Trp53*^*R270H/−*^ mice*) *[58]*.* KPshp53 cells derive from PDAC that developed in a *Pdx1-cre; LSL-Kras*^*G12D*^*; Col1a1-TRE-shp53- shRenilla; Rosa26-CAGs-LSL-rtTA-IRES-mKate2* mice generated by blastocyst injection and maintained on doxycycline chow [59]*.* All the cell lines were grown in DMEM supplemented with 10% FBS (Gibco) and penicillin–streptomycin (2U/mL) at 37°C, 5% CO_2_. KPshp53 were maintained in 1 μg/ml doxycycline to keep off p53 through doxy-dependent shRNA against Trp53; to allow p53 expression the doxycycline was removed 72 h before further procedure; KPshp53 were propagated on collagen-coated plates (PurCol, Advanced Biomatrix, 0.1 mg/ml).

siRNA transfection was performed with Lipofectamine™ RNAiMAX (Invitrogen, Thermo Fisher Scientific), using a pre-designed siRNA targeting mouse Trp53 (Silencer™ Select Pre-Designed siRNA 20 nmol by ThermoFisher Scientific, cat. no. 4390815) at a concentration of 50 nM, and as a control, 50 nM control siRNA (Silencer™ Select Negative Control No. 1 siRNA 40 nmol by ThermoFisher Scientific, cat. no. 4390844) was used for comparison.

### Metabolomic analysis

For MS-based metabolomic analysis, five biological replicates for control KPC^R270H^ or KPC^R172H^ cells and p53-depleted cells were collected after 72 h of silencing. A total amount of 1 × 10^6^ per condition were pelleted, snap-frozen and stored at − 80°C. Samples were processed using an Ultra-high-pressure liquid chromatography (UHCPL) coupled to high-resolution tandem mass spectrometry (MS/ MS, Vanquish and QExactive, Thermo Fisher, San Jose, USA). Methods were previously described [60].

Metabolic pathway enrichment analysis (‘Pathway Analysis’) was performed with MetaboAnalyst 5.0 web-based software (www.metaboanalyst.ca) using metabolites that from the metabolomics were significantly differentially expressed (−1.3 < Fold change < 1.3, *P* < 0.05) in mouse PDAC p53^R172H^ proficient-/ deficient-cells.

### ATAC-seq and RNA-seq

For ATAC-seq, KPC^R270H^ cells were transfected as previously described [15]. Briefly, a total amount of 100.000 cells were pelleted and resuspended using a cryopreservation solution with 50% FBS, 40% completed DMEM and 10% DMSO. Following snap-frozen step, samples were stored at − 80°C.

For the RNA-seq, total RNA was extracted as reported below. Collected samples for ATAC-seq and RNA-seq were processed by Active Motif. ATAC-seq Peak-calling alignment and reads mapping to the mouse genome was performed using UCSC Genome Browser (http://genome.ucsc.edu/index.html) and Integrated Genome Browser (http:// bioviz.org).

### ChIP-seq analyses

The ChIP-seq analysis was performed by ChIP-Atlas Database (https://chip-atlas.org/peak_browser) and Integrative Genomics Viewer (http://www.broadinstitute.org/igv/) for peaks visualization: H3K4me1 murine pancreas (id: SRX4928874), H3K4me1 murine PDAC (id: SRX2853283), H3K4me3 murine pancreas (id: SRX3262436), H3K27ac murine PDAC (id: SRX2853270), ATAC-seq murine pancreas (id: SRX17578977), ATAC-seq murine PDAC (id: SRX8893869), ATAC-seq N6 (id: SRX2852935), ATAC-seq N5 (id: SRX2852934), ATAC-seq P3 (id: SRX2852937), ATAC-seq P2 (id: SRX2852936), ATAC-seq T6 (id: SRX2852939), ATAC-seq T3 (id: SRX2852938), ATAC-seq M3P (id: SRX2852942), ATAC-seq M1L (id: SRX2852941), H3K27ac human pancreas (id: SRX10187637), H3K27ac human PDAC (id: SRX2853313).

### RT-qPCR

Total RNA was isolated from KPC cells using RNeasy Mini Kit (Qiagen) and reverse transcribed with SensiFAST cDNA Synthesis Kit (Meridian Bioscience, BIO-65054) according to the manufacturers’ protocols. Real-time PCR (RT-qPCR) was performed using Fast SYBR Green PCR Master Mix (Applied Biosystems). Primers for the amplification are following indicated: mGapdh Forward 5’- CCTCGTCCCGTAGACAAAATG- 3’, mGapdh Reverse 5’-TCTCCACTTTGCCACTGCAA-3’, mTrp53 Forward 5’-TGAAACGCCGACCTATCCTTA-3’, mTrp53 Reverse 5-’GGCACAAACACGAACCTCAAA-3’, Lamin B1 Forward 5’-CTTCGCTCTTTGTGCGGTAG-3’, Lamin B1 Reverse 5’-GGGACCGAAGGTACAAACCA-3’, Slc3a2 Forward 5’-TAAAAACATCACCCCTGCCTC-3’, Slc3a2 Reverse 5’-GGGAGGAGCAAAAGAGATCAG-3’, Mouse CkB Fwd 5’-CAGACTGGCGTAGACAATCC-3’, Mouse CkB Rev 5’-AGGTTGTCTGGGTTGAGGTC-3’, Mouse Arg2 Fwd 5’-GTGTATCCTCGTTCAGTGGGC-3’, Mouse Arg2 Rev 5’-ATGAGCATCAACCCAGATGACA-3’.

All results were normalized to the mouse housekeeping gene Glyceraldehyde 3-phosphate dehydrogenase (GAPDH) and the relative gene expression was calculated with the 2 − ΔΔct method.

### Seahorse stress test

Seahorse XF^e^24 Flux analyzer was used to assess mitochondrial function in KPC270 cells. On day 1 cells were seeded in 6cm^2^ dishes at 300.000 density in DMEM medium, supplemented as described in cell culture section. On day 2 cells were transfected as in siRNA transfection section.

On day 4 transfected cells were re-seeded onto Seahorse XF24 cell culture microplates (Agilent, 100,777–004 V7-PS TC-treated) at the density of 20.000 cells/well; on the same day, XF24 sensor cartridge was hydrated with Seahorse XF calibrant (Agilent, 100840–000) and incubated in non-CO_2_ incubator at 37°C.

On day 5, cell medium was replaced by using in 500 μL/well of XF DMEM (Agilent, 103,575–100) supplemented with 18 mM d-glucose (Merck, G7021), 1 mM of sodium pyruvate (Merck, P5281) and 2 mM of l-glutamine (Merk, G8540). Cells were incubated in non-CO_2_ incubator at 37°C for 45 min before the assay was performed.

Oxygen consumption rates (OCR) was measured by using XF^e^24 Extracellular Flux Analyzer. Sequentially injection of oxidative stress modulators [1 μM oligomycin (Merck, O4876), 1.5 μM carbonyl cyanide-4-(trifluoromethoxy)phenylhydrazone (FCCP) (Merck, C2920) and a mix of 0.5 μM rotenone (Merck, R8875) and 0.5 μM antimycin A (Merck, A8674)] was used to provide information on production of ATP, maximal respiration rate, mitochondrial spare capacity and glycolysis. OCR was measured as previously described [61].

Measurements were normalised on cell number by 1 µg/ml H-33342 staining for 30 min at 37°C to identify nuclei (365 ± 50/535 ± 45 nm); 25 fields/well were imaged at 20 × magnification (2 × 2 pixel binning) by using an automated microscope (Array-Scan VTI HCS Reader, Cellomics, PA) and an imaging software (vHCS SCAN, Thermo Fisher).

### Bioinformatic and mutational signature analysis

mRNA expression analysis for LamB1, Slc3a1, ARG2 and CKB was retrieved from the Pancreatic adenocarcinoma dataset (TCGA Pancancer) by interrogating the cBioPortal database (25,26). The whole patient cohort (*n* = 184) was divided according to the TP53 mutational status (wild-type (wt) vs mutated (Mut)). To assess the expression of Arg2 and CkB across the different stages of Pancreatic Adenocarcinoma, the GEPIA website was used (http://gepia.cancer-pku.cn/about.html). For Kaplan–Meier overall survival analysis, patient cohorts from PanCancer dataset was divided into two groups on the basis of the gene expression level (Low expression, High expression) by the computational webtool Kaplan–Meier plotter at https://kmplot.com [62].

Mutational signature calls were downloaded from the ICGC Data Portal on the 25th of July 2023. (URL:https://dcc.icgc.org/releases/PCAWG/mutational_signatures/Signatures_in_Samples/SP_Signatures_in_Samples). MAF (masked somatic mutation) files of all publicly available TCGA datasets (33 tumor types) were downloaded from the TCGA data portal on the 27th of July 2023 (https://portal.gdc.cancer.gov) using the TCGAbiolinks R library version 2.24.3 (Colaprico et al., 2015). Tumors were split into three groups according to their p53 mutation type: “R273H mutant” (containing only R273H samples), “other mutants” (all samples with TP53 mutations but not R273H) and “wild type” (samples with no TP53 mutations reported). Statistical significance was assessed with Fisher’s exact test for count data (fisher.test R function). Finally, the plots were produced using R ggplot2 package.

### BH3 profiling

BH3 profiling was developed at the Letai laboratory, and the experiments were performed as previously described [47]. First, single-cell suspensions were stained with the viability marker Zombie Violet (#423,113, BioLegend, Koblenz, Germany), washed with PBS, and resuspended in MEB (150 mM mannitol, 10 mM hepes–KOH pH 7.5, 150 mM KCl, 1 mM EGTA, 1 mM EDTA, 0.1% BSA, 5 mM succinate) in a final volume of 25 μL. Secondly, cell suspensions (3 × 10^4^/ml) were incubated with 0.002% digitonin and deposited in a 96-well plate (#3795, Corning, Madrid, Spain) with increasing concentrations of BIM BH3-only protein (final concentration of 0.1, 0.3, 1, 3, 10, 30 and 100 μM) for 1 h following fixation with formaldehyde. Cells were finally stained with cytochrome C antibody (Alexa Fluor® 647 anti-Cytochrome c, #612,310, BioLegend). Individual BH3 profiling analyses were performed using triplicates for DMSO, alamethicin (#BML-A150-0005, Enzo Life Sciences, Lorrach, Germany), and the different BIM BH3 peptide concentrations used. The expressed values are the average of three readings performed with a high-throughput spectral flow cytometry Cytek AURORA instrument (Cytek Biosciences, California, USA) from the Scientific and Technological Centers of the University of Barcelona. % of cytochrome C released is used to calculate delta priming (%), representing the difference between siRNA/shRNA p53 cells minus control cells for a given peptide.

### Mitochondrial membrane potential

Cells were transfected with control siRNA or Trp53 siRNA and collected 72 h after transfection. Cells were then incubated for 20 min at 37°C to allow the permeabilization of the JC1 dye (0.2 μM; Invitrogen), and analyzed by flow cytometry. Fluorescence signals from JC-1 staining were collected in the relevant fluorescence channels to monitor changes in mitochondrial membrane polarization based on the dye’s structural form. The orange-red fluorescence (~ 590 nm) channel detects J-aggregates, which are present in polarized mitochondria, while the green emission (~ 520 nm) channel captures monomeric JC-1 in the cytoplasm, indicating mitochondrial depolarization. The ratio of 520 nm to 590 nm fluorescence was used to represent shifts in mitochondrial membrane polarization.

### Cell viability and competitive proliferation assays 

For the MTT assay, cells were transfected with control siRNA or Trp53 siRNA. 24 h post transfection, cells were plated in 96 well culture plates (1 × 10^4^ cells/well). The following day, cells were treated with increasing amount of NH4Cl (1 mM, 5 mM, 10 mM, 50 mM, 100 mM). Further, after 24 h of treatment, the medium was removed and 10 µl MTT solution from the stock (5 mg/ml) was added and cells were incubated at 37°C in 5% CO2 incubator in the dark for 1.5 h. The medium was then removed, and Formazan crystals formed by the cells were dissolved using 100 µl of DMSO. The absorbance was read at 540 nm on a multiwell plate reader (TECAN infinite M200 PRO).

For competitive proliferation assays, lentiviruses were produced by co-transfection of 25.106 293FT cells with expression vector (13,5 ug ipUSEPR-sgRNA-RFP-Puro or 13.5 ug pFUGW-lentiCas9-Blast Addgene #52962) and helper vectors (13.5ug pLP1-13.5ug pLP2-4.5ug pLP/VSV6 Invitrogen #K4975-00) using Lipofectamine™ LTX Reagent with PLUS™ Reagent (Invitrogen 15,338,100). Six hrs after transfection the medium was refreshed and the viral supernatant was collected after 48 h and 72 h from transfection, filtered through a 0.45 um filter (Millipore) and supplemented with 10 ug/ml of polybrene (Millipore # TR-1003) before adding to target cells. To conduct competitive proliferation assays, cells were transduced with lentiCas9-Blast and selected using 10ug/ml of blasticidin (Sigma-Aldrich #15,205) to generate stable Cas9-expressing cell lines. These cells were subsequently transduced with ipUSEPR-p53gRNAs to about 50% transduction efficiency. gRNAs targeting Renilla and RPA3 are used as a negative and positive controls, respectively. The percentage of RFP + cells was monitored by Flow cytometry using FACS Canto cytofluorometer (BD Biosciences, Franklin Lakes, NJ, USA). Fluorescence background levels were set with untransduced cells. Experiments were performed independently 3 times.

### Statistical analysis

All statistical analyses were performed, and P values were obtained using the GraphPad Prism software (GraphPad Software Inc.). All results are expressed as the mean ± standard deviation (SD). Statistical significance between groups was calculated by two-tailed Student’s t test or one-way ANOVA. Tests performed with *P* < 0.05 were considered statistically significant. Statistical significance is shown as *P < 0.05, **P < 0.01, ***P < 0.001.

## Supplementary Information

Additional file 1: Supplemental Figure 1. A Western Blot analysis of KPC270 and KPC172 cells following p53 silencing by siRNA transfection. B Analysis of mitochondrial potential by JC1 staining and FACS analysis in KPC270 following p53 silencing and/or treatment with carbonylcyanide m-chlorophenylhydrazone (CCCP). CCCP represents positive control. JC1 ratio (orange-red fluorescence ~590 nm emission / green fluorescence ~520 nm emission) is reported as percentage of the untreated siCTR transfected cells. Graphs are shown as mean ± SD of at least 4 biological replicates. * *p* value <0.05; ** *p* value <0.01. Supplemental Figure 2. Bar plots showing the nucleotide level of p53R172H proficient and deficient PDAC cells. The graphs are shown as mean ± SD of 5 biological replicates. * *p* value <0.05; ** *p* value <0.01; *** *p* value <0.001. Supplemental Figure 3. A Reactome-enrichment plots showing “Glutathione conjugation” and “Biological oxidations” as one of the top enriched pathways upon mutant p53R270H depletion in KPCR270H cells. B Western Blot analysis of KPshRNA cells following removal of doxycycline and restoration of wt p53 expression. C Frequency of SBS mutational signatures co-occurrence / mutual exclusivity in p53 mutant-expressing tumours compared to the p53WT counterpart tumours.

## Data Availability

Available upon requests and relevant datasets will be deposited in public repositories.
