# Supplementary figures and images for: Selective metabolic regulations by p53 mutant variants in pancreatic cancer
Source: J Exp Clin Cancer Res. 2024 Nov 26;43:310. doi: 10.1186/s13046-024-03232-3 (PMC11590503; doi:10.1186/s13046-024-03232-3)

# Supplementary Figure 1

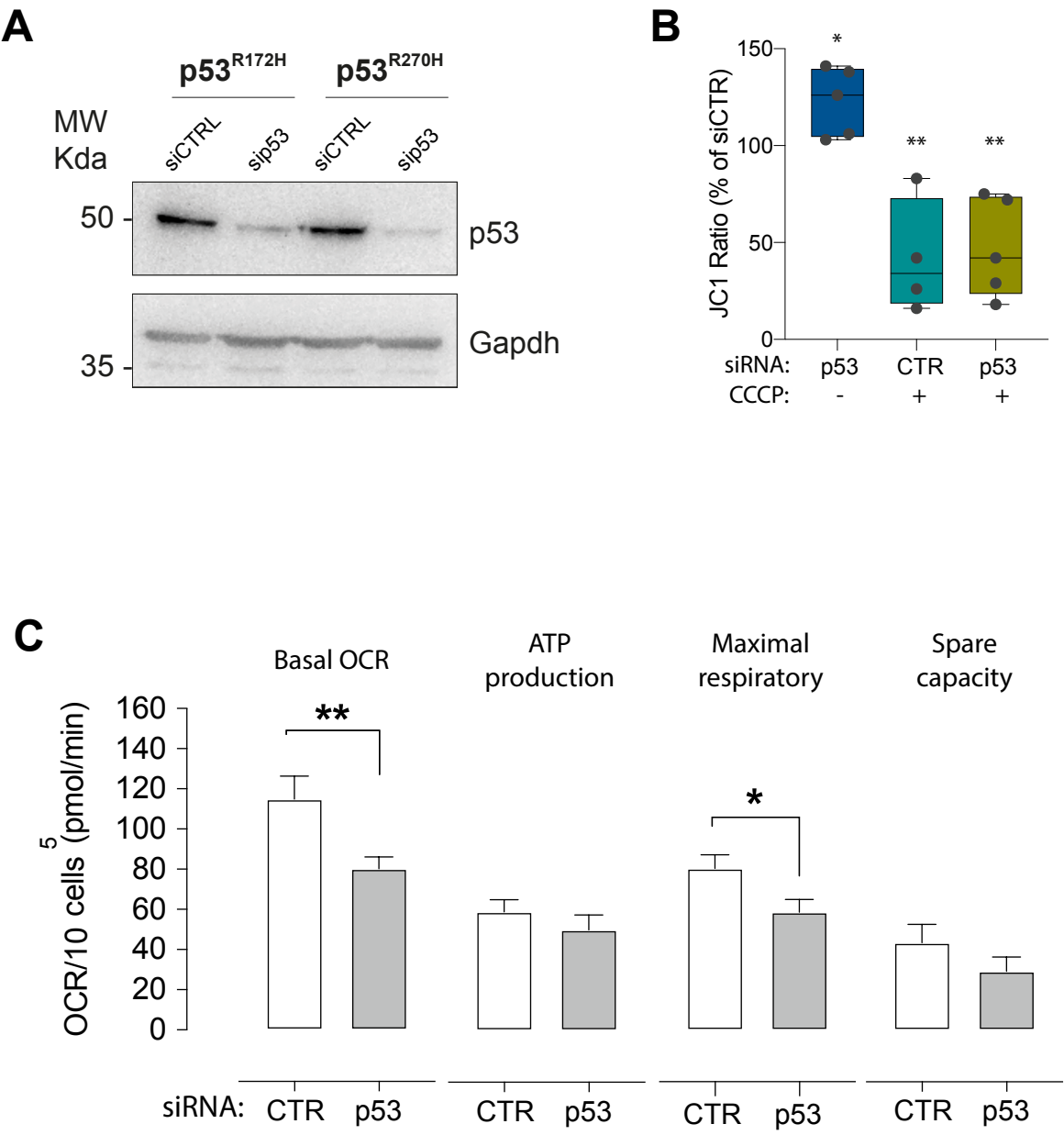

Supp Figure 2

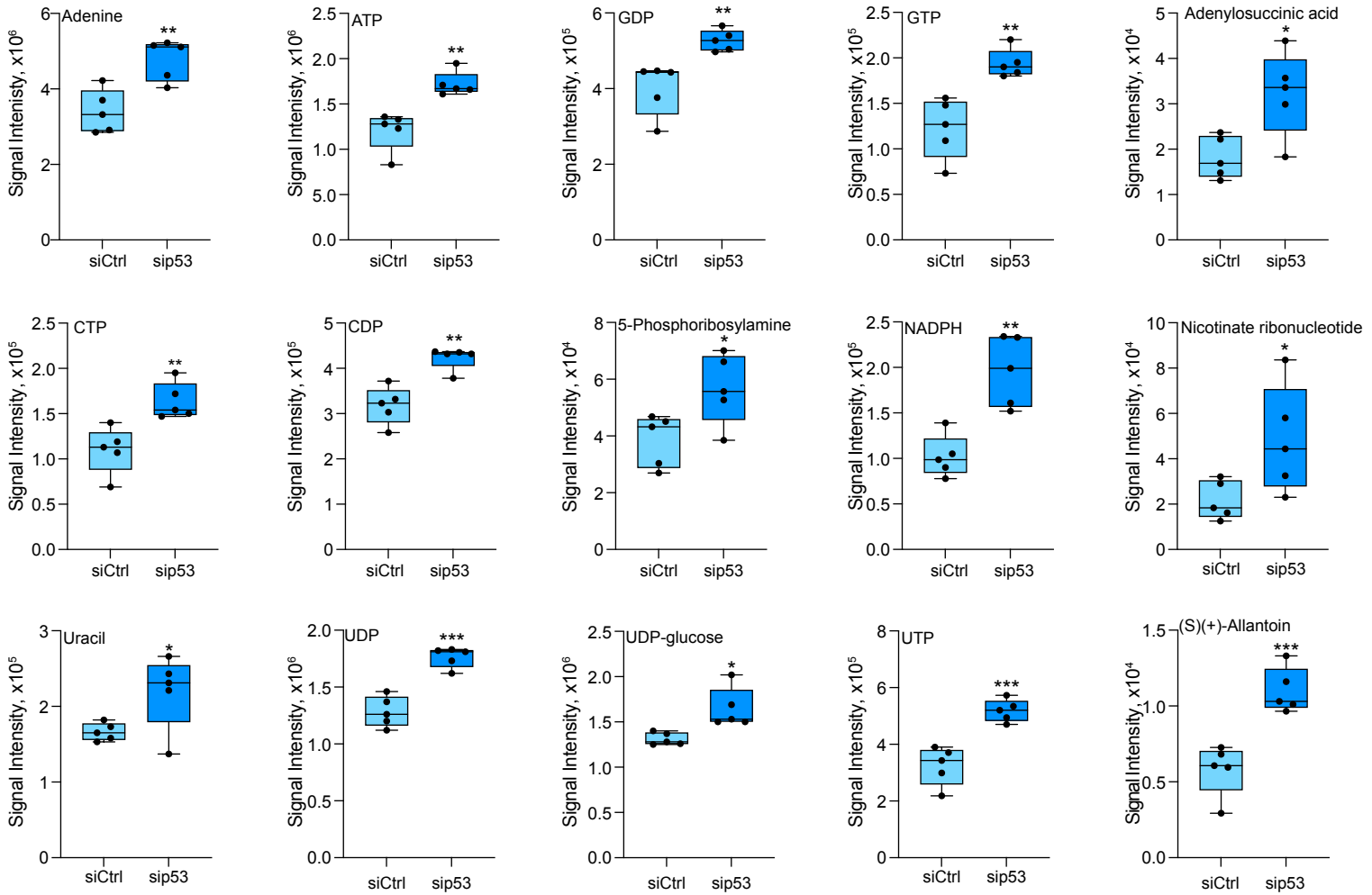

Supplementary Figure 3

A

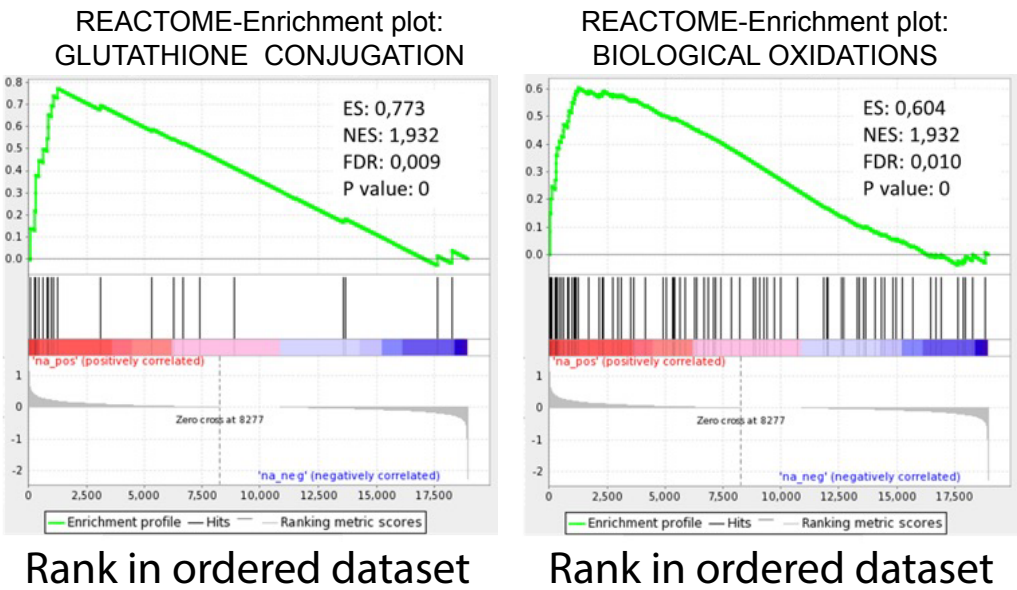

B

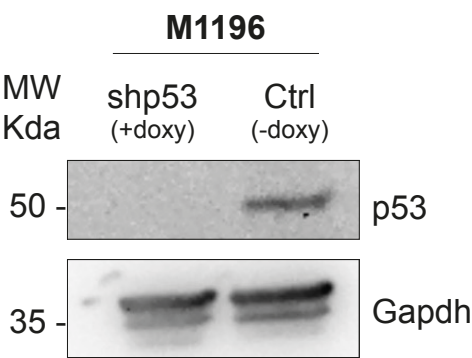

C

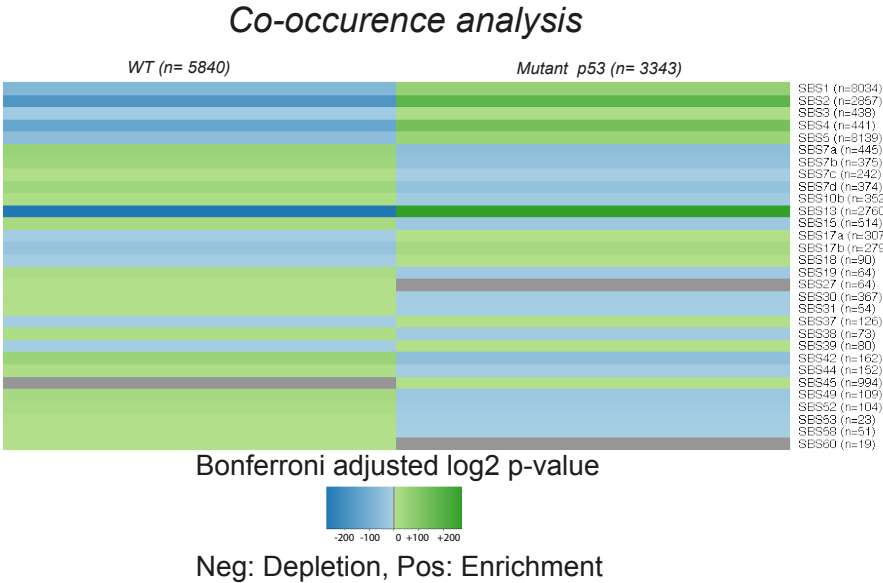

Supplement: Supplementary file 1 — Additional file 1: Supplemental Figure 1. A Western Blot analysis of KPC270 and KPC172 cells following p53 silencing by siRNA transfection. B Analysis of mitochondrial potential by JC1 staining and FACS analysis in KPC270 following p53 silencing and/or treatment with carbonylcyanide m-chlorophenylhydrazone (CCCP). CCCP represents positive control. JC1 ratio (orange-red fluorescence ~590 nm emission / green fluorescence ~520 nm emission) is reported as percentage of the untreated siCTR transfected cells. Graphs are shown as mean ± SD of at least 4 biological replicates. * p value <0.05; ** p value <0.01. Supplemental Figure 2. Bar plots showing the nucleotide level of p53R172H proficient and deficient PDAC cells. The graphs are shown as mean ± SD of 5 biological replicates. * p value <0.05; ** p value <0.01; *** p value <0.001. Supplemental Figure 3. A Reactome-enrichment plots showing “Glutathione conjugation” and “Biological oxidations” as one of the top enriched pathways upon mutant p53R270H depletion in KPCR270H cells. B Western Blot analysis of KPshRNA cells following removal of doxycycline and restoration of wt p53 expression. C Frequency of SBS mutational signatures co-occurrence / mutual exclusivity in p53 mutant-expressing tumours compared to the p53WT counterpart tumours. [file 13046_2024_3232_MOESM1_ESM.pdf]
